# A spatially comprehensive canopy cover dataset derived from NASA’s ice, cloud and land elevation satellite-2 (ICESat-2) for the state of Alabama, USA

**DOI:** 10.1016/j.dib.2025.111902

**Published:** 2025-07-17

**Authors:** Lana L. Narine, Blake Johnson

**Affiliations:** College of Forestry, Wildlife and Environment, Auburn University, 3301 FWS Building, 602 Duncan Drive, Auburn, AL 36849, USA

**Keywords:** ICESat-2, ATL08, Lidar, Satellite, Canopy cover, forests

## Abstract

NASA’s Ice, Cloud, and land Elevation Satellite-2 (ICESat-2) has already demonstrated an extraordinary capability to assess forests, including providing measurements of canopy heights, and estimating aboveground biomass (AGB) and canopy cover. Despite these advancements, the application of the mission’s data to deriving continuous estimates of canopy cover, as is fundamental parameter for assessing forest conditions, is not well-understood. Here, we present a statewide (135,760 km²) canopy cover dataset at a 30 m scale across mixed temperate forests of the southern United States (US), highlighting feasibility of applying ICESat-2 data for deriving canopy cover, and providing a basis for further upscaling. This dataset provides continuous estimates of canopy cover across forests in the state of Alabama, in the southern United States, for the year 2022. Non-forests were masked out of the final dataset, and canopy cover values within each 30 m pixel, range from 0 to 72 %. Only freely and openly available remote sensing data were used to generate this dataset. ICESat-2′s along-track, vegetation product data were acquired and filtered to retain nighttime-only samples within classified forests, calibrated using reference canopy cover from airborne lidar data, and then extrapolated to achieve wall-to-wall coverage, using a Random Forests (RF) model. The mapped output represents a large-area ICESat-2-derived canopy cover product, highlighting applicability of ICESat-2 for canopy cover information and synergistic use with free and open space-based and other available ancillary products for this information. This dataset is openly accessible through the Open Science Framework.

Specifications table

**Table:** 

Subject	Earth & Environmental Sciences
Specific subject area	Canopy cover
Type of data	Raster (Tiff)
Data collection	Ice, Cloud, and land Elevation Satellite-2 vegetation product data (ATL08) acquired in the year 2022, were downloaded from the National Snow and Ice Data Center (https://nsidc.org/home), and used compute canopy cover with established equations [1]. Reference airborne lidar data were retrieved from two National Ecological Observatory Network (NEON) sites, from the NEON data portal at https://data.neonscience.org/, and from US Geological Survey’s 3D Elevation Program for three national forest sites (https://www.usgs.gov/3d-elevation-program). Airborne lidar data for the two NEON sites were acquired in 2023, and data from 3DEP were collected in 2017 and 2018. Sentinel-2 surface reflectance data and topographic predictors were derived from Google Earth Engine at https://developers.google.com/earth-engine/datasets/catalog/sentinel-2 and https://developers.google.com/earth-engine/datasets/catalog/COPERNICUS_DEM_GLO30. Ancillary variables, consisting of National Land Cover Database (NLCD) 30-m land cover and canopy cover products, were downloaded from the National Land Cover Database website, at https://www.mrlc.gov/data. These data were combined, filtered to remove erroneous samples and observations outside of forested areas, and then used for mapping canopy cover for the state of Alabama.
Data source location	• Country: United States • State: Alabama
Data accessibility	Repository name: Open Science Framework Data identification number: 10.17605/OSF.IO/VM68P Direct URL to data: https://osf.io/vm68p/
Related research article	None.

## 1 Value of the Data

• These data are valuable in characterizing forest conditions, including assessing forest health, modeling habitat suitability and estimating other critical forest biophysical attributes. Representing a fundamental metric, canopy cover contributes to the definition of forests (e.g., minimum 10 % canopy cover by the Food and Agriculture Organization of the United Nations (FAO)), it is important in forest inventories and well-demonstrated as an important predictor of other critical forest attributes, like forest canopy height and aboveground biomass.
• The canopy cover dataset can be reused by other researchers as input to other models, including biomass and carbon models, fuel models, and for assessing disturbance (e.g., wildfire, diseases). It provides canopy cover information for the year 2022, derived from the combined use of multi-platform lidar, multispectral satellite imagery and ancillary data. Beyond forestry, the data support research across fields, including ecology, hydrology, and wildlife sciences, including habitat suitability and species distribution modeling.
• The dataset is spatially complete, providing large-area information derived entirely from openly available Earth observations. ICESat-2 and Sentinel-2 are both ongoing missions that provide consistent three-dimensional observations and imagery respectively, enabling repeated application of a cost-effective workflow for updating the baseline dataset and supporting monitoring.

## 2 Background

**Fig. 1:**
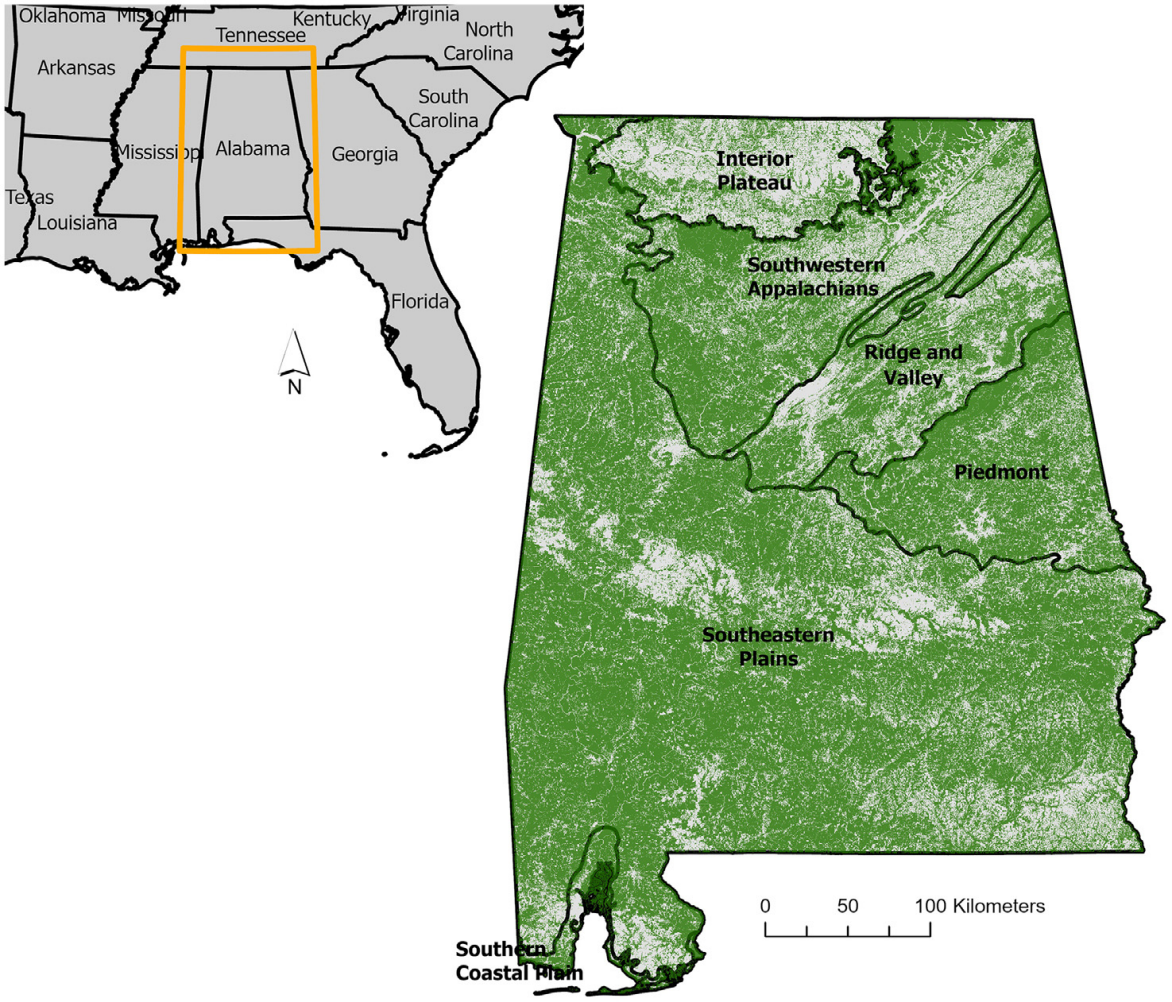
Map of the state of Alabama, located in the southeastern United States, which served as the study area. The region is ecologically diverse, spanning six Level III ecoregions as shown on the larger map of the state [7]. Forests are symbolized in green, based on land cover data from the National Land Cover Database [8].

Since its launch in September 2018, NASA’s Ice, Cloud, and Land Elevation Satellite-2 (ICESat-2) has demonstrated an extraordinary capability to characterize forest attributes. The three-dimensional measurements provided by the mission have enabled mapping of a suite of forest parameters at varying spatial scales, including continental-scale maps of canopy heights (e.g., [2]), local-scale to regional-scale forest aboveground biomass (AGB) products (e.g., [[3], [4], [5]]) and local-scale maps of continuous canopy cover [6]. Despite the demonstrated capability to derive continuous estimates of canopy cover and its significance in assessing forest conditions, ICESat-2-derived canopy cover maps are not yet available for the contiguous United States (U.S.). As a pilot study and precursor to a continental-scale product, a workflow was developed and applied to create this statewide canopy cover dataset. This dataset provides a large-area estimation of continuous canopy cover across temperate forests of Alabama (Fig. 1), in the southern United States, for the year 2022. Much of this region (∼70 %) is forested, spanning across six distinctive ecoregions as illustrated in Fig. 1, and the state is top ranked in terms of biodiversity in the United States. Here, we expanded upon an established ICESat-2 canopy cover framework from earlier work [6] to create a 30-m map across the predominantly forested state. This dataset presents new canopy cover information based on structural measurements from an ongoing spaceborne lidar mission and represents output generated from synergistic use of only openly available sources of data.

## 3 Data Description

**Fig. 2:**
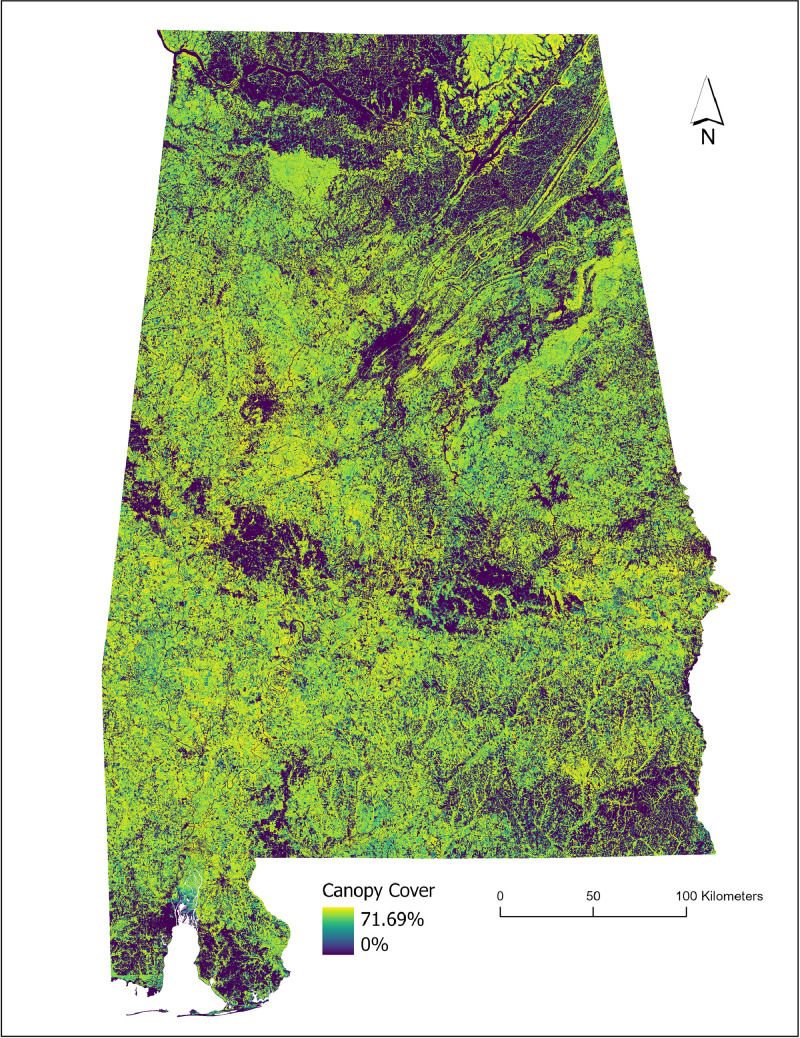
Visualization of the final canopy cover map generated for the state of Alabama.

This paper describes a large-area canopy cover product across temperate forests in the state of Alabama, situated in the southern United States. This dataset (Fig. 2) was created to support research across fields, including forestry, wildlife sciences, and hydrology. In addition, the output supports ongoing efforts to design a continental-scale canopy cover product using data from the Ice, Cloud and land Elevation-2 (ICESat-2) mission. Earlier work highlighted the capability of deriving canopy cover from the mission’s vegetation data product or ATL08 with the development and evaluation of specific equations [1], and the application of 100 m segment estimates to mapping at a 30-m scale in specific forested sites in the southern United States [6]. However, limitations in upscaling from spatially incontiguous ATL08 segments to spatially continuous pixel estimates were noted. Specifically, achieving accurate estimates across heterogeneous forest areas proved challenging, and upscaled pixel-level estimates of canopy cover yielded modest accuracies; R² values of 0.51–0.52, when compared with reference airborne lidar-derived estimates. Furthermore, as the previous methodology was based on site-specific modeling (e.g., a 75 km² site in southern Alabama), there is an urgent need for a workflow specifically designed for large-area mapping. Building upon earlier ICESat-2 canopy cover work, this raster dataset represents results from a pilot study for modeling canopy cover over a broader scale, across an estimated area of 135,760 km². The canopy cover dataset and corresponding map of uncertainty have been uploaded to a free and open platform, the Open Science Framework repository. The forest canopy cover product is a 30 m raster dataset, where each pixel contains percent cover as a continuous estimate for the year 2022. A forest/non-forest mask generated from the 2021 National Land Cover Database (NLCD) land cover product, was used to mask out (pixels set to 0) non-forested areas in the final product to ensure representation of classified forests (forests, shrubs forested or forested wetlands or NLCD classes = 41–52, 90, and all other categories were reclassified to non-forest).

**Fig. 3:**
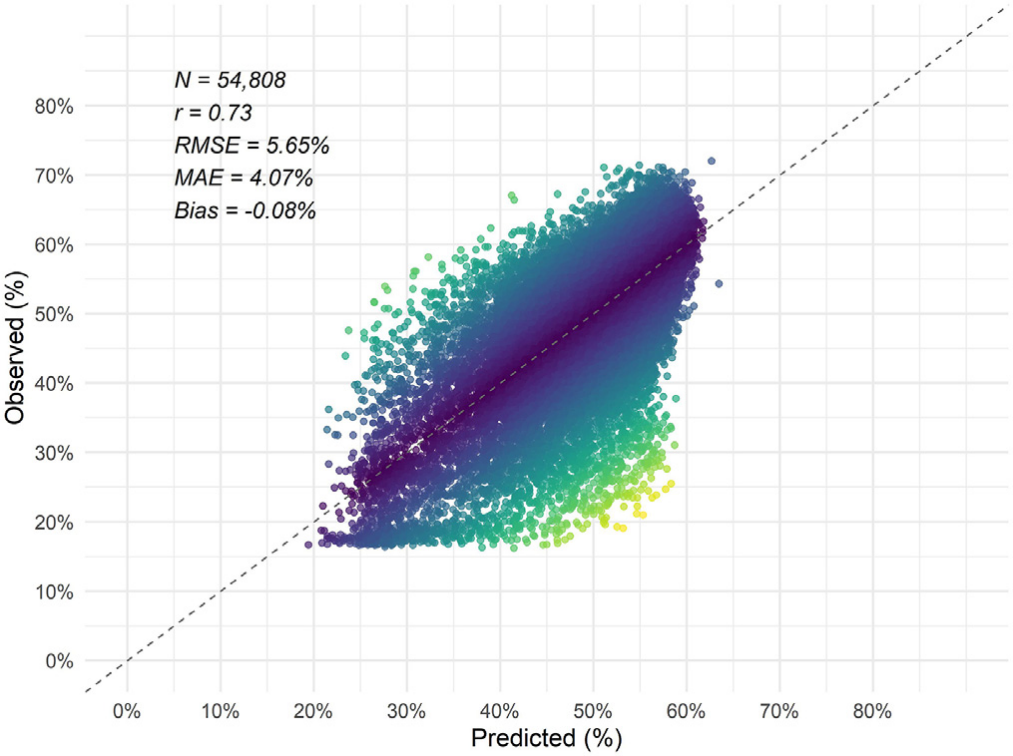
Scatterplot of observed versus predicted canopy cover using test data, with the 1:1 line shown. Points are colored by absolute error using a viridis color scale, with yellow indicating higher error and darker colors indicating lower error.

Overall, the final predictive model for canopy cover exhibited low bias with moderate performance in estimating canopy cover. Based on error metrics calculated using approximately 30 % of samples (*N* = 54,808) withheld as test data, the canopy cover model produced a correlation coefficient (r) of 0.73, with a negative bias of −0.08 % and root mean square error of 5.6 % (Fig. 3).

## 4 Experimental Design, Materials and Methods

**Fig. 4:**
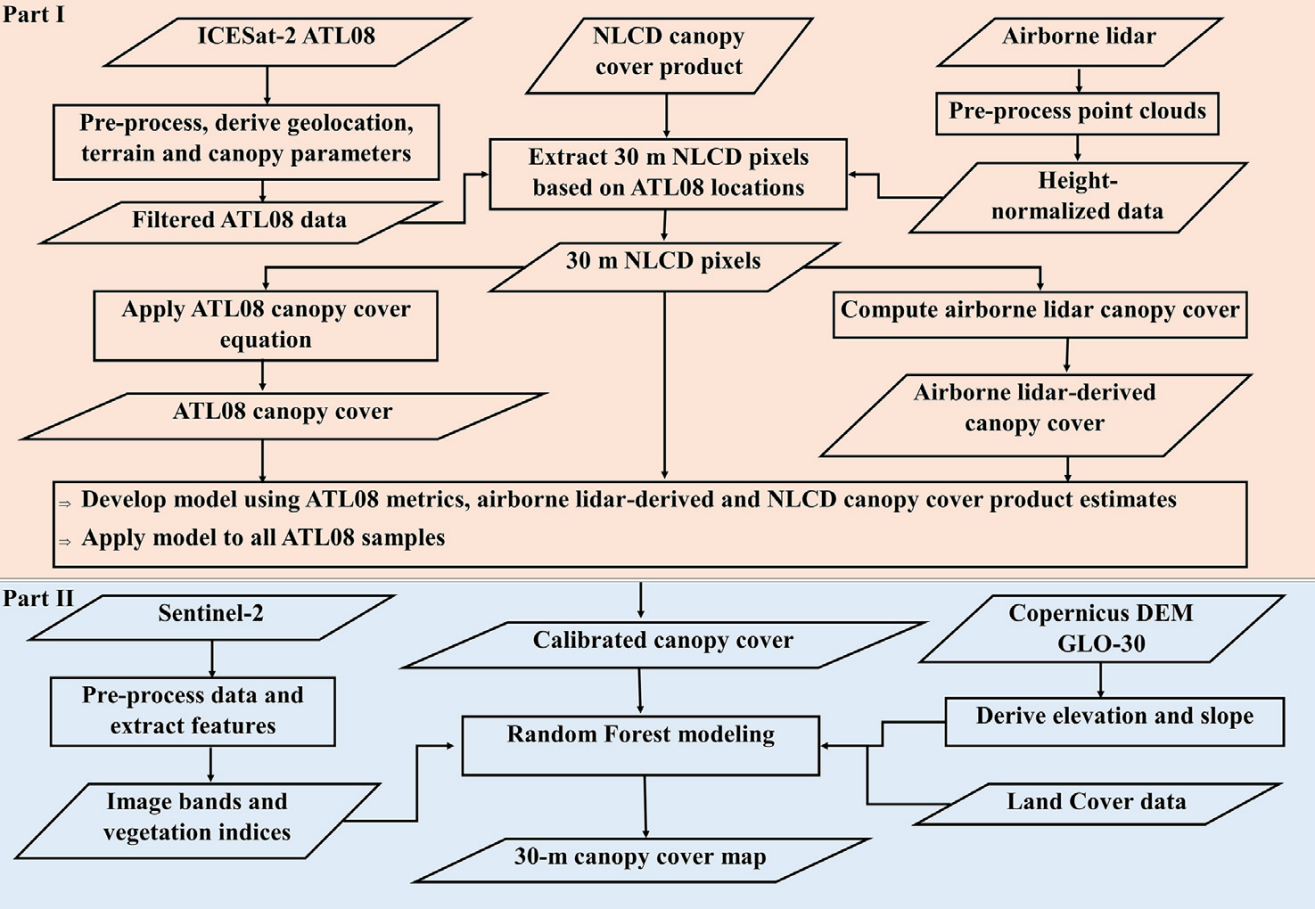
Methodological workflow for generating gridded canopy cover. Part I was carried out using data for five sites within the state of Alabama (Talladega National Forest, Dead Lake, Conecuh National Forest, Bankhead National Forest, Tuskegee National Forest). Part II used ATL08 samples and wall-to-wall predictors across the state of Alabama.

The workflow for creating the canopy cover dataset is shown in Fig 4. and described in the following sub-sections. To create the 2022 canopy cover dataset, the selection of ICESat-2 and Sentinel-2 data was limited to 2022, and airborne lidar data acquired as close as possible to that year were utilized. In the first part of the methods shown in Fig 4. (Part I), canopy cover was computed from processed airborne lidar point clouds, and separately, from filtered ICESat-2 vegetation product data (ATL08). Using a subset of the data, a regression model was developed using airborne lidar canopy cover as the response variable, and ATL08-derived canopy cover and NLCD TCC as predictors, which was subsequently applied to all ATL08 samples. In Part II, as shown in the figure, spatially incontiguous ATL08-estimated canopy cover estimates were then extrapolated using a suite of wall-to-wall predictors with a Random Forest model, to create the 30 m dataset.

### 4.1 Reference canopy cover

Reference canopy cover data were derived from freely available, open-access datasets provided by the National Ecological Observatory Network (NEON) and United States Geological Survey 3D Elevation Program. NEON, operated by Battelle, provides ecological data in discrete sites across the continental United States, to support research in diverse ecosystems and surfaces, including terrestrial and aquatic ecosystems. The most recent airborne lidar point clouds, acquired in 2023 from two NEON sites in Alabama, were retrieved to develop references for canopy cover. The coordinate reference system used for the datasets was EPSG:32,616, corresponding to WGS 84 / UTM Zone 16 N. The NEON sites were Dead Lake (32.54173, −87.80388), and Talladega National Forest (32.95047, −87.39326). Airborne lidar datasets from 3D Elevation Program (3DEP) were also downloaded for three national forest sites: Conecuh National Forest (31.1359, −86.5967), acquired in 2018, Bankhead National Forest (34.237221, −87.334442), acquired in 2017 and Tuskegee National Forest (32.4853, −85.5592), acquired in 2017, for better representation of forest conditions in the state. To maintain consistency with the spatial resolution of existing Landsat-based tree canopy cover products from the NLCD and spatial resolution analyzed in prior investigations (e.g., [1]), airborne lidar-derived canopy cover estimates were generated at the 30 m pixel extent. All airborne lidar point clouds were height-normalized using the lidR R package [9] and height-normalized airborne lidar point clouds were used to derive canopy cover, as the percentage of returns above 2 m within a NLCD pixel.

### 4.2 ICESat-2-derived canopy cover

ICESat-2 uses a photon-counting lidar system operating at 532 nm, the Advanced Topographic Laser Altimeter System (ATLAS), which is affected by noise from the atmosphere and solar background [10]. Findings from terrain and canopy validation studies suggest use of night acquisitions for vegetation, which have a considerably lower noise rate than day acquisitions [11,10]. Nighttime acquisitions of ICESat-2 data acquired for the year 2022 were retrieved from the National Snow and Ice Data Center. The mission’s dedicated vegetation product data, ATL08, containing along-track terrain and canopy estimates reported in fixed 100 m segments, were selected for this work. ATL08 segments were filtered according to recommendations from related vegetation studies [[10], [11], [12]], to use nighttime acquisitions from strong beams, within reasonable height ranges (<45 m, based on h_canopy values). Using the filtered dataset, canopy cover was computed as the percentage of canopy and top-of-canopy of total canopy, top-of-canopy and ground photons as follows:

$$\frac{\left(n\_ca\_photons\;+\;n\_toc\_photons\right)}{\left(n\_ca\_photons\;+\;n\_toc\_photons\;+n\_te\_photons\right)}\times \;100$$

Using segment geolocation information, the data were further filtered based on spatial coincidence with NLCD land cover, to retain forest samples. While a total of 182,703 samples were retained across the state, a subset of the data that coincided with the reference airborne lidar-derived canopy cover (*n* = 14,960) from the discrete sites (NEON and 3DEP) were further integrated with NLCD tree canopy cover (TCC) to develop a linear regression model predicting airborne lidar canopy cover as a function of ATL08-derived canopy height, canopy cover and TCC. The model, using 98th percentile height (h_canopy or RH98) from ATL08, ICESat-2-derived canopy cover and TCC was then applied to all ATL08 samples across the state as a corrected measure of canopy cover.

### 4.3 Upscaling of ICESat-2-derived canopy cover

Satellite imagery, imagery-derived vegetation indices and land cover information are demonstrated to be effective predictors in mapping forest attributes with spatially discrete ICESat-2 observations [2,5,6]. A suite of wall-to-wall predictor variables was used to train a Random Forest model, with the updated ICESat-2-derived canopy cover as the response. Predictors were Sentinel-2 surface reflectance bands (12 image bands) resampled to 30 m using bilinear interpolation, spectral indices computed from the image bands (Normalized Difference Vegetation Index, Enhanced Vegetation Index, Modified Normalized Difference Water Index, Normalized Difference Built-up Index, and Bare soil index), the Copernicus GLO-30 digital elevation model (DEM) and DEM-derived slope, and NLCD land cover. The Sentinel-2 surface reflectance data were derived and processed in Google Earth Engine (GEE) to generate an annual cloud-free median composite for the year 2022, and elevation and slope were also derived from the DEM in GEE. The predictors were combined with the ICESat-2 response, split intro training and test sets (70/30) and then used for model building, evaluation and mapping with Random Forest (RF). ModelMap, an R package for RF model building and map generation [13], was used, and the resulting model was applied to gridded predictors to generate the mapped dataset. A corresponding map of uncertainty, computed as the standard deviation of the individual-tree predictions for every canopy cover pixel, was also generated.

## Limitations

Free and open datasets were leveraged for the analyses, and, at the time of the study, the most temporally aligned data available were utilized. While the ICESat-2 lidar data and satellite imagery were temporally consistent, both from the year 2022, temporal mismatches exist between the airborne lidar and the satellite observations. To limit the impact of tree growth over time, airborne lidar data selection was restricted to acquisitions from 2017 or later.

## Ethics Statement

Authors confirm that ethical requirements have been read and followed for publication in Data in Brief. The current work does not involve human subjects, animal experiments or any data collected from social media platforms.

## CRediT authorship contribution statement

**Lana L. Narine:** Conceptualization, Investigation, Methodology, Writing – original draft. **Blake Johnson:** Methodology.

## Declaration of Competing Interest

The authors declare that they have no known competing financial interests or personal relationships that could have appeared to influence the work reported in this paper.

## Data Availability

The Open Science FrameworkAn ICESat-2-derived Canopy Cover Map for Alabama, USA (Original data).

## Appendix Supplementary Materials

Image, application 1
